# Evidence for a Behaviourally Measurable Perseverance Trait in Humans

**DOI:** 10.3390/bs11090123

**Published:** 2021-09-09

**Authors:** Ilmari Määttänen, Emilia Makkonen, Markus Jokela, Johanna Närväinen, Julius Väliaho, Vilja Seppälä, Julia Kylmälä, Pentti Henttonen

**Affiliations:** 1 Department of Psychology and Logopedics, Faculty of Medicine, University of Helsinki, 00014 Helsinki, Finland; emilia.makkonen@helsinki.fi (E.M.); markus.jokela@helsinki.fi (M.J.); julius.valiaho@gmail.com (J.V.); vilja.seppala@helsinki.fi (V.S.); julia.kylmala@helsinki.fi (J.K.); pentti.henttonen@helsinki.fi (P.H.); 2 Cognitive Science, Department of Digital Humanities, Faculty of Arts, University of Helsinki, 00014 Helsinki, Finland; 3 VTT Technical Research Centre of Finland Ltd., 70211 Kuopio, Finland; johanna.narvainen@vtt.fi

**Keywords:** behavioural persistence, behavioural perseverance, cold pressor test, hand grip endurance, verbal reasoning, anagram

## Abstract

The aim was to create and study a possible behavioural measure for trait(s) in humans that reflect the ability and motivation to continue an unpleasant behaviour, i.e., behavioural perseverance or persistence (BP). We utilised six different tasks with 54 subjects to measure the possible BP trait(s): cold pressor task, hand grip endurance task, impossible anagram task, impossible verbal reasoning task, thread and needle task, and boring video task. The task performances formed two BP factors. Together, the two-factor solution is responsible for the common variance constituting 37.3% of the total variance in the performances i.e., performance times. Excluding the impossible anagram task, the performance in any given task was better explained by performances in the other tasks (i.e., “trait”, η^2^ range = 0.131–0.253) than by the rank order variable (“depletion”, i.e., getting tired from the previous tasks, η2 range = 0–0.096).

## Highlights

- Behavioural perseverance or persistence (BP) of humans can be measured behaviourally.
- BP possibly forms a two-factor structure.
- BP traits are a better predictor of performance than depletion of individuals’ personal resources (i.e., getting tired) in a task.

## 1. Introduction

Previous research has made some attempts to study human behavioural perseverance or persistence (abbreviated in this study generically as “BP”) in multifaceted behavioural experiments. Much of the research seems to be rather old (for a review, see Feather [1]). Examples of ways to measure persistence behaviourally include Hartshorne, May, and Mailer [2], Crutcher [3], and Rethlingshafer [4]. The mentioned studies included several different ways to possibly measure persistence and to correlate them with each other. These tasks include multi-story resistance, puzzle mastery, paper and pencil puzzle solution, fatigue and boredom in mental work, hunting for hidden objects, continued standing on the right foot, eating cracker and whistling, and solving a toy puzzle. Crutcher [3] found some evidence for general persistence factor among school children (tests included card-house building, mechanical puzzle solution, addition, picture copying, and cancelling As), and found there to be a 0.3 correlation between persistence and intelligence. In fact, rather than behavioural persistence, factor analysis has more popularly been used to study the structure of cognitive skills [5]. On the other hand Eysenck measured persistence behaviourally by a single physical endurance task: holding one leg above an adjacent chair [6,7]. In addition, there are some recent studies about possible behavioural perseverance [8].

Typically, the mentioned studies measured persistence by time spent in a difficult task. A less common method has been to count the number of attempted (impossible) trials. However, the early studies seemed to lack a concentrated effort to rigorously find out whether certain tasks would be more suitable than others in measuring persistence or perseverance at least in some contexts. There seems to be a lack of consensus regarding if and how different measures are strongly associated with each other. In addition, the term that should be used for these kinds of tasks is not clear and depends partially on the linguistic background of the researchers. We have opted to use perseverance and persistence, which we aimed to measure without referring to any specific questionnaire or theory and is abbreviated in this study as “BP”. Despite the similarity of the concepts, many consider that there is a difference between the meaning of perseverance and persistence. For instance, perseverance sometimes refers to struggling toward a goal more than persistence does. As the tasks used in the study were behavioural, we tried to avoid too lengthy lexical speculation.

BP-related behaviour may be relevant for real-life situations. Mask wearing induces perception of mental fatigue [9]. On the other hand, psychological inflexibility was not associated with academic performance [10]. It is an open question as to how BP would be reflected in real-life behaviour or outcomes.

It has been suggested that persistence should be understood as a motivational phenomenon rather than a psychological trait [1]. Some researchers have emphasised persistence or self-control as a certain type of limited resource, or that the behaviour is depleted during a difficult task, although in a different context than in this study [11]. In this study, we used “depletion”, i.e., the poorer performance in later tasks due to depleted mental or other personal resources, as an alternative explanation for performance. Depletion could also be simply described as “getting tired”.

Some of the tasks used in this study had inspiration from previous research. We used unsolvable anagrams as a possible measure of BP in this study. In a study of the effect of emotional intelligence on stress response, Matthews et al. [12] utilised the impossible anagram-solving task as one of the stress conditions. The task included only unsolvable anagrams, whereas the current study used both solvable and unsolvable anagrams. Segerstrom and Nes [13] used both solvable and unsolvable anagrams as a measure of persistence after a self-control demanding task. In a study of self-control, Casa de Calvo and Reich [14] presented early-semester and late-semester students with sets of both impossible and possible anagrams in an effort to assess whether the latter group had less patience with the task, i.e., indicating some type of “depletion” of mental resources. Some articles have criticised anagram studies that found a self-control-related depletion effect [15], while others have dismissed this criticism [16]. In any case, according to some of the literature reviewed above, the “depletion effect” was postulated to explain the persistence in the anagram tasks. Thus, we took the “depletion effect” as one possible competing explanation in predicting the task performance.

One of our possible measures of BP was a “boring video” task. In the task, subjects faced the decision of trying to achieve a better performance by continuing to watch the video in which nothing happens or quitting the task. It was presented as the final task. There is at least one previous study that used some version of the “boring video” task [17]. Some previous research has also used some type of handgrip endurance task, although not in the same way as in this study [18]. The cold pressor test (here referred as “task”) is a well-known experimental layout in psychology [19,20,21] that is often used to measure blood pressure and heart rate reactivity and may reflect pain tolerance, among other traits. Despite its typical use in other contexts and its use in measuring pain tolerance, in this study, we used the cold pressor task as a possible measure of BP.

The aim of the study was to investigate whether people have trait-like BP, or more specifically, a behaviourally measurable ability and willingness to persist and continue to pursue an adverse, difficult, and unpleasant task. We created, to our best knowledge, a completely new experimental layout in the laboratory to study this possible trait or set of traits. It is likely that related concepts, which include perseverance and persistence, may have differences between different languages and countries. For instance, most English-language literature consider only positive aspects of persistence and perseverance, whereas they may carry also negative or maladaptive features in some other languages, such as in Finnish [22]. The common feature of the different experimental tasks used in this study was their hypothesised demand for a trait of being able or willing to continue performing an unpleasant task. Alternatively, the performances could be more or less uncorrelated, or the depletion of the mental resources during the experiment could be the strongest predictor of the performances. According to our best knowledge, this is the first time this kind of design has been attempted.

We utilised six different tasks that were, to our perception, at least in some way unpleasant or arduous to perform. The tasks included physical discomfort, frustrating motor task, cognitive challenge, and commitment to a boring task. We selected a one time-variable from each task: the total performance time for most of the tasks and the time used for the first impossible puzzle or anagram in the corresponding two tasks. It should be noted that the tasks were quite different from each other and the possible associations between different task performances would not be self-evident.

As it has been previously found that personality traits correlate with stress physiology [23], it may be relevant to analyse ECG as well as variables related to body size. Thus, we included some additional measures of stress physiology to our analyses in order to figure out if the possible BP trait or traits would be strongly associated with stress reactivity or even possibly caused by it. If the associations between stress physiology and BP trait(s) were very high, further investigation on the relationship would be warranted.

As the study was explorative, no strict hypotheses were presented, but the major study questions were the following:

- Is there evidence for a trait-like feature (BP) in the subjects’ performances? I.e., are the performances, measured by performance time used in a difficult or unpleasant situation, between different tasks intercorrelated?
- Is there evidence for “depletion” of mental resources when the subjects have to continue adverse, difficult, or unpleasant tasks?
- Do previous tasks “deplete” or influence the performance of the later tasks negatively?
- Does the effort made in a subtask “deplete” or influence the performance in the subtasks negatively within the same task?
- Is the performance in the other tasks a stronger predictor of performance in a given task (“BP trait effect”) than the rank order in which the subject is conducting the task (“depletion effect”)?
- Are the BP traits (i.e., factor or factor-based sum score variable(s)) associated with “biological” variables, i.e., sex, age, height, weight, or stress reactivity?

## 2. Materials and Methods

### 2.1. Measurement Devices and Computer Programs

All measurement devices were from the manufacturer BIOPAC. The computer program AcqKnowledge 5.0.2 (BIOPAC Systems Inc., Goleta, CA, USA) was used to collect physical measurement data. Presentation 20.1 (Neurobehavioral Systems Inc., Albany, CA, USA) was used to present the tasks and questions to participants and to collect data describing their performance.

### 2.2. Experimental Design and Layout

#### Design and Pre-Preparation of the Experimental Layout

Before starting the experimental phase of the study, great effort was made to design and preliminarily test the different tasks to investigate whether or not they would work with subjects. Available students and other suitable, healthy people participated as subjects in the preliminary tests. All of the researchers and research assistants that carried out the further experiments had also tried all of the tasks themselves before starting to test other people.

Possible problems in the different experimental layouts included floor and ceiling effects (i.e., it was intended that the task performances would not get very high or very low values too often). In addition, the tasks were designed in a way that the frustration and willingness to quit increases over time.

The time limits of the tasks and task difficulty were adjusted by the preliminary tests. In the cold pressor task, different water temperatures were tested to ensure that most of the subjects would give up in a limited amount of time. In the hand grip endurance task, several ways of conducting the experiment were considered, including using the dynamometer in the actual task, but that would have not guaranteed similar task difficulty for different participants. Based on the performances in the preliminary tests, strength categories were created so that each subject would experience similar difficulty levels. (This goal was achieved; correlations between hand grip strength and the performance in hand grip endurance tasks were non-significant and without a statistical trend, *p* > 0.1.).

In the thread and needle task, several different preliminary set-ups were tried. In the end, an optimal way to conduct the task was found in which the individuals did not realise immediately that the task was impossible. The task instructions also had to be made in a way that ensured this was the case.

In the anagram and verbal reasoning tasks, the difficulty level was roughly evaluated in the preliminary tests, and based on this information, a pseudorandomisation of the task order was made.

In the boring video task, careful preparation based on the preliminary tests was made to ensure that most of the subjects would look at the video at least some short amount of time, but it was made boring enough so that almost all of the subjects would give up after some amount of time. In the video, there was a movement of a person in a white lab coat in 10 s after the beginning of the video in order to show the subjects it was not a video loop.

In general, as can be seen from Table 1, there are no floor effects: all of the subjects tried all of the tasks at least some amount of time, and each of the participants also successfully solved at least one anagram and at least one verbal reasoning puzzle. There were some subjects that used all of the given time, but the average performance as well as overwhelming majority of the performances were much lower, as can be inferred from the standard deviation.

Some other tasks were considered in the preliminary testing phase, but these were not chosen into the study. The purpose was to select such tasks that worked in a more or less linear fashion in all of the subjects, i.e., their willingness to continue decreases with time. Thus, a task utilising moving chess pieces with boxing gloves was not included, although it was tried in some preliminary tests: the subjects reported getting more motivated as they got better in the supposedly frustrating task. This was true even when the task was started all over again after any chess piece fell down.

### 2.3. Recruitment

Participants were recruited to the laboratory from those that had answered an online questionnaire that was sent to university mailing lists. At the recruitment stage, subjects that suffered from diagnosed chronic heart disease, diagnosed type 1 diabetes or clinical depression, or were on medication that affected the central nervous system were excluded and thus not invited to the laboratory. This type of exclusion criteria was considered sufficient and has been used in our studies previously. No sign of mental health issues among the recruited was observed with the exception of one depressed subject, who reported their mental health status only after the experiment and was then excluded from the data. In addition, when starting the laboratory tests, a question concerning hand health was asked from each subject in order to guarantee the safety of the tasks. As a result of the invitation method (university mailing lists), the sample is skewed toward a more educated population.

### 2.4. Execution of the Tasks

A total of 55 subjects participated in the laboratory phase of the study. Despite our best efforts of pre-screening, one subject was excluded after finishing all of the tasks because of self-reported depression and very poor performance in the tasks. Descriptive statistics of the laboratory subjects can be seen in Table 1.

When the subjects arrived at the laboratory, they were first instructed to wash their hands. Then, they filled out an informed consent form and a form concerning their hand health. The dominant hand was used as the active hand to perform tasks if it was healthy. If there were any major or recent health issues with the dominant hand, the non-dominant hand was used as the active hand. Altogether, two subjects used their non-dominant hand.

Physiological measures included continuous blood pressure, electrodermal activity (EDA), electrocardiogram (ECG), respiration, and electromyogram (EMG). Performance and self-reported psychological variables were also measured in all of the tasks.

Participants performed six tasks: cold pressor task, handgrip endurance task (two times), verbal reasoning task, anagram task, thread and needle task, and boring video task. In the beginning, there was a rest phase of five minutes for baseline measurement of physiological signals. Each task was followed by a break of two minutes, excluding the last task.

The task order was partly pseudorandomised before each subject arrived. The structure was the same for all of the participants: Physically challenging tasks were never followed by another physically challenging task, and the video task was always performed last. Participants also assessed their performance and mood before and after each task. The handgrip endurance task was performed twice in a row, but in most analyses (with the exception of Question 2), only the first trial was used, as the performances highly correlated with each other (r = 0.813, *p* < 0.0001).

Participants performed each task alone in a separate room. They were given text instructions via computer screen and answered when needed using a keyboard. Participants had one minute to prepare for each task after instructions before performance. The researcher monitored the participant with a camera in a separate room. In some tasks, the researcher entered the room to bring tools and clock tasks.

The participants were given two movie tickets each for their participation.

### 2.5. Tasks

#### Cold Pressor Task

The cold pressor task is commonly used and has been described previously [19,20]. In this task, the participants were asked to place their hand in ice-cold water so that the water reached their wrist. The time to reach the pain threshold was measured first. The participants were instructed to immerse the hand and to pull it out as soon as they felt pain. After the performance, they were instructed to warm their hand in warm water for a short while.

In the second stage, participants were told to keep their hand in the container as long as they could. They were instructed to give oral estimates from 0–10 of their subjective level of pain as they were performing the task. The researcher was in the room with the participant during the performance and used a keyboard to log the vocal estimates.

Unless the subject withdrew the hand earlier, the immersion was discontinued by the researcher after three minutes. After the performance, the participants were instructed to warm their hand in warm water for a short while.

The water was held in a 7 L container with ice and circulated using a pump (EHEIM compactON 300). Ice was added in the container approximately 30 min before the participant entered the laboratory. The temperature of the water was typically just below one degree Celsius (M = 0.77 °C, SD = 0.56 °C). The amount of ice needed was determined in the preliminary study phase.

The level of performance in the subsequent analyses was measured by how long the participants could keep their hand in the cold water container.

### 2.6. Hand Grip Endurance Task

**Figure 1:**
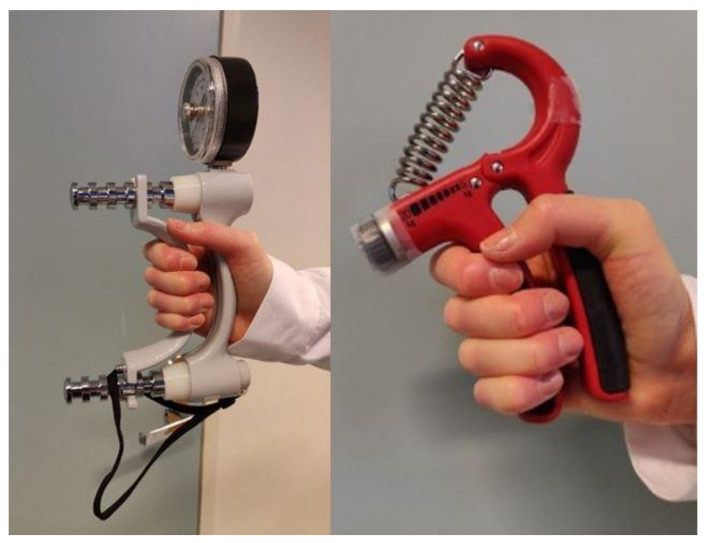
Hand grip strength measurement device (**left**) and hand grip endurance task device (**right**).

The hand grip endurance task was designed for this study and was not based on previously known task designs. In this task, the maximum hand grip strength was first measured with a dynamometer (Figure 1). The dynamometer could not be used in the hand grip endurance task, as the amount of force the participant has to use cannot be controlled. In the dynamometer, there is no upper limit. Based on the performance, a hand grip exercise tool was chosen from an appropriate category to make the task equally difficult for all of the subjects. The categories were formed based on the preliminary testing. The categories were not significantly associated with the performance time.

Maximum strength, category:

<21 kg: category 1;
21–27.5 kg: category 2;
27.6–37.9 kg: category 3;
38–47.9 kg: category 4;
>48 kg: category 5.

In the second stage, participants were instructed to clench the hand grip exercise tool in their fist and maintain the grip as long as they could (Figure 1). They were instructed to give oral estimates from 0–10 of their subjective level of discomfort as they were performing the task. The researcher was in the room with the participant during the performance and used a keyboard to log the oral estimates. If the participant was still maintaining the grip, the performance was discontinued by the researcher after three minutes.

After the performance, participants were instructed to repeat the task and given one minute to prepare for the upcoming task.

The level of performance in the subsequent analysis was indicated by how long the participants could maintain the grip.

### 2.7. Verbal Reasoning Task

A verbal reasoning task was designed for this study and was not based on previously known task designs. In this task, participants were instructed to solve verbal reasoning tasks or puzzles presented in Finnish on the computer screen. They were told that the tasks had three difficulty levels: “easy”, “slightly more challenging”, and ”hard”. The verbal reasoning tasks had been assigned to the categories based on the amount of information they contained. Unbeknownst to the participants, the four point “hard” verbal reasoning tasks were actually impossible to solve.

The following is an example of an “easy” verbal reasoning puzzle (the example is in English, to accommodate the reader):

*“Easy: 1 point.*
*You have three red balls, three blue balls and a red coffee cup. What is the colour of items you have the most?*
*(A) Red  (B)  Blue  (C) I have equal amount of both”*
Right answer: A

The following is an example of a “slightly more challenging” verbal reasoning puzzle:

*“Slightly more challenging: 2 points.*
*Five boxes are arranged in a row. One of the boxes contains a ball. The ball is not in a box that has only one box next to it. The first box is closer to the third box than it is to the box that contains the ball. In which box is the ball in?*
*(A) First     (B)   Second     (C)   Third    (D)   Fourth    (E)   Fifth”*
Right answer: D

The following is an example of an “impossible” verbal reasoning puzzle (the participant was not aware that it was impossible):

*“Hard: 4 points;*
*Timo tells you that his birthday is on one of the following days:*
(A) May 15 (B) May 16 (C) May 19
(D) June 17 (E) June 18
(F) July 14 (G) July 15 (H) July 17
(I) August 14 (J) August 15 (K) August 17
*Timo tells only the month to Seppo, and only the number of the day to Anna.*
*Seppo: I do not know when Timo’s birthday is, but I do know that Anna does not know either.*
*Anna: At first I did not know when Timo’s birthday is, but now I do.*
*Seppo: Then I also know when Timo’s birthday is.*
*When is Timo’s birthday?”*
Note: there is no correct answer as the task was designed to be impossible.

Participants were told that by solving these verbal reasoning puzzles they could earn one, two, or four points depending on the difficulty level. They used one hand and a keyboard to give their answers. After instructions, participants were presented with an example task.

Participants were presented with verbal reasoning puzzles from different difficulty categories. This order was the same for all participants, but the tasks were pseudorandomised inside the difficulty categories. The order was: E S I E I S S E S E S E S S (easy = E, slightly more challenging = S, impossible = I).

Participants had seven minutes to complete as many tasks they were able to, and they were allowed to skip verbal reasoning tasks if they so wished. The participants were informed that they should solve as many verbal reasoning puzzles as possible and collect as many “points” as possible. The time participants spent on the impossible puzzles was recorded.

As different individuals differ in their capacity to solve verbal reasoning puzzles and solving a verbal reasoning puzzle per se does not reflect a higher BP, the level of “performance” in the subsequent analysis was indicated by the duration the participants attempted to solve the first impossible verbal reasoning puzzle.

### 2.8. Anagram Task

The anagram task was designed for this study and was not based on previously known task designs. In this task, participants were instructed to form new words from the letters of words presented on the computer screen. Participants used a keyboard to give their answers. The task was implemented in Finnish. After instructions, participants were presented with an example anagram OLUT (“beer”). This was used for practice. The right answer was TULO (“income”).

Anagrams in the actual task had six letters. Some had multiple, and some had a single solution. Based on the performances in the preliminary study phase, the anagrams were assigned to three difficulty categories: easy, hard, and impossible. Participants were not informed about these categories, and one point was given regardless of the category of the solved anagram.

The following is an example of the type of anagrams used (the example is in English to accommodate the reader):

CALLER

Right answer (for example): RECALL

Participants were presented with anagrams from different difficulty categories. They were not aware of the difficulty categories. This order was the same for all participants, but the order of the specific anagrams was pseudorandomised inside the difficulty categories. The order was as follows: E E I E I E I E H E H E H E E E H H; (easy = E, hard = H, impossible = I).

Participants had five minutes to solve as many anagrams as possible, and they were able to skip anagrams if they wished. Unbeknownst to them, three of the anagrams were impossible to solve. The participants were informed that the amount of anagrams solved would measure their level of achievement in the task.

As different individuals differ in their capacity to solve anagrams and solving an anagram per se does not reflect a higher BP, the level of “performance” in the subsequent analysis was indicated by the duration the participants attempted to solve the first impossible anagram.

### 2.9. Thread and Needle Task

The thread and needle task was designed for this study and was not based on previously known task designs. In the thread and needle task, participants were instructed to get a thread through the eye of a needle. Participants were not told in advance that this task was supposed to be frustrating. However, the width of the thread made the task impossible to perform, especially given the time limit and the instructions, according to which the subject could not touch the thread with their mouth or the other hand and could only hold the thread beyond a knot approximately 5 cm from the tip of the thread.

Participants were instructed to press a button on the keyboard when they had completed the task or when they wanted to end the task. The time participants used before pressing the button was measured. Instructions to end the task were automatically given on the computer screen five minutes after the beginning of the task.

The level of “performance” in the subsequent analysis was indicated by how long the participants continued to attempt to put the thread into the eye of the needle.

### 2.10. Boring Video Task

The boring video task was designed for this study and was not based on previously known task designs. The video task was always the last task in order to ensure that “tricking” the participants would not discourage them from the other tasks. In this task, participants were instructed to watch a video. They were told that they could stop watching the video by pressing a button when they felt they were ready to answer questions about its events.

In the video, a white lab coat is moving in air flow to indicate to the participant that it indeed is a video and not a picture. After approximately 10 s, a person with a white lab coat moves across the screen to indicate that the video is not a loop.

Questions were never asked, but the length of time for which the participant watched the video was measured. The task was automatically discontinued after five minutes.

The level of “performance” in the subsequent analysis was indicated by the duration the participants continued to watch the boring video.

## 3. Data Analysis

### 3.1. ECG Data-Analysis

ECG data were collected using a modified Lead II electrode placement with a sample rate of 2000 Hz. Frequencies below 0.05 Hz and above 35 Hz were removed using second order Butterworth filters, along with a 50 Hz notch filter for mains noise. R-peaks were detected using the algorithm in ECGlab toolbox for MATLAB (v 8.1; [24]). After the detection of R-spikes, resultant data were visually inspected according to Porges and Byrne [25] for falsely detected and ectopic beats. Ectopic values were interpolated for heart rate variability (HRV) calculations. Interbeat interval (IBI) and values describing HRV were calculated from the R-peak series. Consecutive IBI values were transformed into uniformly sampled 4 Hz series using cubic spline interpolation.

Average IBI, maximum IBI, and minimum IBI were calculated per each differing experimental task or phase. Calculated HRV values were following: high frequency (HF; 0.15–0.4 Hz) and low frequency (LF; 0.08–0.15 Hz). These mean square power values were obtained from the interpolated IBI series using the Welch method with 256-point Hanning windows with 50% overlap.

### 3.2. Factor Analysis

One time-variable was selected from each of the following tasks to be used in the factor analysis: a thread and needle task, a cold pressor task, an impossible anagram task, an impossible verbal reasoning task, a hand grip endurance task, and a boring video task. The time-variable to represent the “performance” in each of the tasks was selected by the following criteria: the overall time spend attempting the task was used when applicable. In two cases (impossible verbal reasoning task and anagram task), this variable was not useful, as the tasks had a constant running time and a different internal logic. Instead of overall time, the time used for the first impossible subtask was used, as all of the subjects were exposed to that particular subtask and were not running out of time by then. All variables used in factor analysis were tested for skewness and kurtosis. The impossible verbal reasoning task, impossible anagram task, and thread and needle task were log transformed in order to get skewness and kurtosis below the threshold of 2, as is recommended for factor analysis.

Factor analysis was conducted by Maximum Likelihood as the extraction method (Table 2 and Supplementary Table S1). Both solutions were Varimax-rotated with Kaiser Normalisation.

### 3.3. Correlations

Pearson correlations were used in the analyses instead of rank correlations, because they better reflected the internal dynamics in a given task. People are “competing” against themselves: they do not know what kind of performances the other subjects may have. Using rank analyses may hide some of the internal dynamics within a task. Nevertheless, using rank-based methods (Sperman’s rho correlation) provided relatively similar results (data not shown).

### 3.4. Selection of Variables

As noted elsewhere, only one time-variable was selected for the analysis per task. However, other variables were considered, such as the time after 8/10 pain or discomfort in physical tasks.

According to our analyses, there was a high correlation between the complete time and the time after 8/10 level pain in the cold pressor task (r = 0.86, *p* < 0.001). The same was true for the hand grip endurance task (first task: r = 0.627, *p* < 0.001 and r = 0.616, *p* < 0.001). Thus, the complete time of the task and time after 8/10 pain or discomfort were measuring essentially the same thing. However, there may still be reasons to believe that continuation after pain or discomfort is a better measure of BP, and this could be useful when designing future studies with the same rationale.

### 3.5. Sum Variables

Sum variables that could be described as “physical” and “mental” BP traits were created based on the results of the factor analyses in order to evaluate their usefulness. Variables that were formed by adding together two variables were normalised before addition. In addition, the impossible verbal reasoning task and thread and needle task were log transformed before summing them.

## 4. Results

### 4.1. Descriptives

There were 47 women and seven men in the sample. Descriptives can be found in Table 1. Descriptives for IBI variables can be found in Supplementary Table S1.

### 4.2. Question 1: Evidence for Trait-Like Features Based on the Performances

Factor analysis was used to answer this question (see Table 2 and Supplementary Table S1). One time-related variable from each task was selected: total time in other tasks and the first impossible verbal reasoning task time and first impossible anagram task time in the corresponding tasks. There were no great differences between two different factor analysis extraction methods that were tried. Principal Axis Factoring and Maximum Likelihood methods were used in the factor analysis and revealed no relevant differences with each other. Then, the Maximum Likelihood method was used in the final analyses. Both of the methods revealed a two-factor solution: one “physical” trait factor and one “mental” BP trait factor. Two sum variables were formed to represent the two factors by adding together the top two variables from each factor, i.e., the cold pressor task and hand grip endurance task on the “physical sum” variable and the verbal reasoning task and thread and needle task on the “mental sum” variable.

Together, the two-factor solution is responsible for the common variance constituting 37.3% of the total variance in the performances i.e., performance times (see Table 3).

Correlations between the variables created based on the factors and sum variables are shown in Table 4. According to the correlations, it seems that both of the factor variable pairs as well as the sum variables based on them show similar correlations. There were several correlations between different performance variables. In addition, the “boring video task” and “impossible anagram task” were correlated with several other variables, even though they loaded less to factors 1 and 2 and were not included in the sum variables.

No confidence intervals (CI) are presented for correlations in this study, but they are given the final sample size of 54: when correlation is 0.862, 95% CI is from 0.77 to 0.92. When correlation is 0.813, 95% CI is from 0.7 to 0.89. When correlation is 0.721, 95% CI is from 0.56 to 0.83. When correlation is 0.697, 95% CI is from 0.53 to 0.81. When correlation is 0.584, 95% CI is from 0.37 to 0.74. When correlation is 0.495, 95% CI is from 0.26 to 0.67. When correlation is 0.400, 95% CI is from 0.15 to 0.6. When correlation is 0.308, 95% CI is from 0.04 to 0.53. When correlation is 0.273, 95% CI is from 0.01 to 0.5.

### 4.3. Question 2: Evidence for Mental Resource Depletion

To test a possible “depletion” effect, i.e., lower ability or willingness to go on after a previous effort has been made, correlation analyses were conducted with repeated trials in impossible anagram and hand grip endurance tasks.

In the case of anagrams, most of the subjects had enough time to complete the tasks all the way until the third impossible anagram (seventh anagram, when also solvable anagrams are counted). Time used on the first impossible anagram had rather high correlations with the second (r = 0.697, *p* < 0.0001) and third (r = 0.695, *p* < 0.0001) impossible anagrams. The second impossible anagram had a very high correlation with the third impossible anagram (r = 0.857, *p* < 0.0001). The hand grip endurance task also had two similar trials in a row. The correlation between the first and second hand grip endurance task trial was positive and very high as well (r = 0.813, *p* < 0.0001). It is important to note that (1) the directions of the associations are positive, not negative, and (2) the associations are very strong.

It was also analysed whether each of the performance variables were associated with their corresponding “rank order” variable (here, large values indicate the early rank order of the task). Most of the performances did not have any association with their corresponding rank order, even though it could be imagined that the subjects made a greater effort on tasks that were in the beginning of the experiment. There were some exceptions: the cold pressor task performance was slightly associated with its rank order, the earlier the better (r = 0.309; *p* = 0.029). Perhaps surprisingly, the early cold pressor task increased the performance in the thread and needle task (r = 0.289; *p* = 0.042) and decreased the performance in the anagram task (r = 0.286; *p* = 0.044). The early anagram task improved the effort made to solve impossible anagrams (r = 0.327; *p* = 0.02) and predicted better performance in the hand grip endurance task (r = 0.283; *p* = 0.046). The early verbal reasoning task predicted worse performance in the cold pressor task (r = −0.328; *p* = 0.02) and hand grip endurance task (r = −0.312; *p* = 0.029), although there was no association in the second trial of the hand grip endurance task. It must be noted that the significance levels of these findings associated with rank order were rather low, and thus, the associations might not be very reliable.

### 4.4. Question 3: Comparison of Evidence between “BP Trait” vs. “Depletion” Explanation of Performance

In order to compare the relevance between effects of “BP trait” vs. “depletion” of different variables on performance, a separate ANCOVA was made for each of the variables. Results of the analyses can be seen in Table 5. A new variable was made for each analysis separately by summing together all of the other performance variables (standardised), and this was used as an independent variable in the analysis (i.e., the performance in the cold pressor task was explained by the performance in all of the other tasks minus the cold pressor task itself and the rank order of cold pressor task in the subject). Typically, *η^2^* > 0.14 is considered large, *η^2^* ≈0.06 is considered medium, and *η^2^* ≈0.01 is considered small, which suggests relatively large effects for “BP trait” explanation of performance [26].

“BP trait” seems to consistently outperform the “depletion” variable as an explanation of performance. There is only one exception: the impossible anagram task.

### 4.5. Question 4a: Physical Attributes and Task Performance Variables

To understand whether the traits can be explained by physical attributes of the individuals, several variables were analysed. Age, sex, and height did not have any associations, besides the obvious ones (men are significantly stronger, weight is strongly correlated with height, etc.). Maximum hand grip strength was associated with physical sum (i.e., cold pressor task and hand grip endurance task), although not very strongly (r = 0.313; *p* = 0.046), see Supplementary Table S2. Maximum hand grip strength was also associated with body size variables, especially with weight (r = 0.444; *p* = 0.004). Maximum hand grip strength was also positively correlated with age, perhaps because the study population was rather young. It is important to note that hand grip strength was not significantly associated with the first or second hand grip endurance task, indicating that the categorisation and selection of the pre-prepared hand grip endurance devices to different strength levels of the subjects was successful. Hand grip strength was slightly correlated with the performance in the cold pressor task (partial correlation adjusted by sex: r = 0.319, *p* = 0.042).

### 4.6. Question 4b: Physiological Stress Measured by Interbeat Interval (IBI) and HRV

The following results are also relevant for question 3: Do people who have better ability to persist in the tasks differ from other people in terms of their stress reactivity? For instance, is it possible that something “protects” them from adverse effects of the tasks? In these analyses, interbeat interval (IBI) was used as a measure of basic stress, as it reflects both sympathetic and parasympathetic activity. High IBI corresponds to low heart rate, and it indicates both lower sympathetic and higher parasympathetic activity.

Descriptives of IBI variables can be seen in Supplementary Table S3. It is not surprising that the hand grip endurance task has the lowest mean IBI (i.e., the highest heart rate), as it is physically the most active task. The boring video task, on the other hand, has the highest IBI, which also reflects the task in which the individual is just watching a video screen. According to t-tests, the cold pressor task, hand grip endurance task, and boring video task differed from each other as well as from all the other tasks significantly (*p* < 0.05) in terms of IBI. There were some tasks that did not differ from each other: the anagram task IBI did not differ from the verbal reasoning task IBI and from the thread and needle task IBI, and the same is true for the separate analyses on the verbal reasoning task IBI and thread and needle task IBI, which showed the same pattern.

Intercorrelations between different IBI variables are very high, as can be seen in Supplementary Table S4. It can be argued that the IBIs of different tasks are not reflecting separate phenomena but rather reflect the general stress reactivity of the central nervous system. It seems that the intercorrelations between physically less demanding tasks correlate more strongly (r > 0.9) with each other than the physically demanding tasks with each other (r = 0.671). This may reflect the stronger physiological stress in the physically demanding tasks, which can also be seen in Supplementary Table S3.

Supplementary Table S5 reveals the correlations between factor variables, trait variables that were created by summing two variables that best reflected a factor, and IBI variables. It must be noted that IBI variables have a consistent positive correlation with the Factor 1 variable and “Physical BP” trait-sum variables, whereas they have no associations with the Factor 2 variable and “Mental BP” trait-sum variables.

Similar correlation analysis was made with high-frequency (HF) and low-frequency (LF) heart rate variability, but the associations were not significant (data not shown).

## 5. Discussion

We made an experimental behavioural design to study the ability to persist in an unpleasant, difficult, or adverse situation. The aim was to learn whether or not performances in different behavioural measures are correlated with each other and form a factor structure, i.e., behavioural perseverance or persistence (referred as BP in this article).

According to our results, two factors were found: “physical” and “mental” BP factors. The cold pressor task and hand grip endurance task were strongly loaded to the physical factor. The verbal reasoning task was strongly loaded to the mental factor. The thread and needle task loaded rather highly to both factors and slightly more to the mental factor. The boring video task was somewhat loaded to both factors and slightly more to the mental factor. The impossible anagram task was significantly but not very strongly loaded to the mental factor. Different factoring methods did not considerably affect the factor structure. The evidence for the “physical” BP factor is clearly stronger than the evidence for the “mental” BP factor.

A BP trait seems to explain a large part of individuals’ performance in the tasks, as performance in one task could be predicted by the performance in the other tasks. We found some, although not very strong, evidence for the alternative “mental depletion” hypothesis, i.e., the assumption that after an effort has been made, people would deplete or lose their ability to persist in an unpleasant or adverse situation. There was actually some evidence even for a “reverse-depletion” effect. Correlations between the times used on different impossible anagrams revealed that the subjects that used more time in one impossible anagram were also much more likely to use more time in the other impossible anagrams. This is surprising not only because participants get more tired while performing a task but also because the task had a limited maximum time. The same was true for the first and second try on the hand grip endurance task. In that task, there is no evidence that the efforts of the subjects would be “depleted”: participants who had persisted longer in their first try also persisted longer in their second try. On the other hand, there was slight evidence that in some of the tasks, such as the cold pressor task, task order plays some type of role. However, when analysed simultaneously with “BP traits”, the effect size and thus relevance of “depletion” is much smaller.

There is some previous evidence of the so-called depletion effect or “ego depletion”, i.e., tendency in which the participants are unable to carry on doing the same arduous tasks. Ego depletion is typically tested using a sequential task experimenting paradigm, in which a self-control requiring task is repeated. A recent study has estimated the size of the effect, if it exists, to be rather small [27]. This study did not per se use the same theoretical framework, but our findings suggest that some “depletion” occurs when task order is analysed. According to our results, it is clear that the task order has a smaller effect than the hypothesised BP trait(s).

We found only limited “biological” correlates with task performance. Maximum hand grip strength was correlated with the performance in the cold pressor task but not very strongly. Lower heart rate in most tasks was correlated with “physical” BP. Neither HF-HRV nor LF-HRV were associated with factor or sum variables. High IBI has been suggested to be a reflection of stress resilience [28] and is associated with good physical condition [29]. Based on our results, it is not yet possible to say whether the “biological” correlates were based on some kind of causal physiological or psychological effects—for instance, through lower stress sensitivity or better physical condition. Nevertheless, future studies should try to disentangle the possible effect of physical condition on the BP performance.

The early discussion about studying persistence by behavioural means seemed to be mostly replaced by speculation and studies in motivation and “the ability to motivate oneself” in a difficult task [1]. However, it is not clear to us why motivation would be the best, only, or even the strongest explanation in this context. In some other contexts, the contribution of motivation is not emphasised as strongly. For instance, one does not regard cognitive tests as not useful, despite the fact that in order to succeed in them, one has to have the proper motivation. Motivation naturally has an important effect in any study measuring behaviour in a laboratory context, but we argue that this does not make measuring skills or behavioural tendencies useless.

Can persistence or perseverance be too high so that it becomes maladaptive in real-life contexts—for instance, inflexibility in cognitive tasks? It is possible, but at least one study did not find evidence of different psychological inflexibility between low vs. high academic performers [10]. The topic of BP and/or depletion is relevant also in present challenges. For instance, there is evidence that real-life tasks, such as wearing a mask for lengthened periods of time, may lead to perception of mental fatigue [9].

We want to encourage further research with this study. It seems that the most robust finding is the rather strong association between the performance in the cold pressor task and hand grip endurance task. According to our study, it is possible to develop meaningful tests for BP, but one must consider the following issues when designing new tasks: (1) No large floor or ceiling effects should be found in the subjects’ performances. (2) The task difficulty must be adjusted so that most of the subjects’ performances are not near the bottom or top of the scale. Most of the performances should fall more or less in the middle. (3) The task has to have a roughly linear effect on all of the subjects; i.e., no development of skills or other ways of increasing performance should be allowed in the design of the task. This is recommended in order to avoid measuring the development of a skill or motivation rather than BP. (4) The possible differences in subjects’ skills that could influence the performance should be reduced by either experimental design (i.e. making categories based on strength) or by using a homogenous study group (i.e. people with roughly similar educational level). (5) Also practical limitations, such as time and space, should be carefully considered. It would most likely be impractical to develop task designs that would require several hours or large spaces.

There were some limitations in the study. The sample size was relatively small, so more research, especially with larger samples, is needed to replicate the findings. Especially, factor analysis should be performed with larger datasets. More specifically, the results concerning the “mental” BP factor were not strong. It appears that the strongest association was between the two possibly painful physical tasks. On the other hand, there were reasons to believe that the different tasks may have something in common, as the performance in one task was relatively well predicted by the performance in all of the other tasks. This is surprising, because the time-variables that were used to assess performance in fact measured several rather different challenges: to continue a task while knowing it would lead to better performance and discomfort (cold pressor task, hand grip endurance task) vs. to continue a difficult task while not knowing whether or not it would lead to improvement of the result (all the other tasks). It would be beneficial for future studies to disentangle the different requirements in different tasks and to uncover the possible effect of cognitive strategies and other similar complicating factors. Some of the results may be sensitive to the exact set-up that has been created. If this study would inspire later studies, an interesting field of research might arise. Other limitations include the sex and education-level distribution: as the majority of the participants were young female students, it is not clear how well the findings can be extrapolated to the rest of society. For instance, the attitude towards cognitively demanding tasks may differ based on the level of education of the individuals, and possibly some other forms of the tasks should be developed. On the other hand, physical strength, in which there is the largest difference between sexes, had no major role in the performances.

It is easy to understand that the BP trait(s) has huge evolutionary and adaptive implications: it is relevant to learn about the variation between humans when faced with extraordinary hardships or extraordinarily difficult tasks. The results may also have significance beyond human psychology. Our results suggest that evolutionarily relevant personality-like traits can be measured behaviourally in a laboratory setting. The non-verbal parts of the study may create insights on how to develop measures that could be used to measure behavioural traits in both humans and non-human animals. In addition, using ability-like behavioural measures, rather than self-reported measures or other ways of tracking average behaviour, may have benefits in human research too.

## 6. Conclusions

Based on the results, it can be argued that the tasks’ performance, measured by a time variable in each of the arduous tasks, are correlated with each other and form two BP-related factors, roughly “physical” and “mental” BP, the latter being weaker and less clear. There is some evidence of a “depletion” effect, but it is small compared to the “BP trait” effect we postulated, i.e., task performance can be best predicted by the performance in the other tasks.

## Figures and Tables

**Table 1:** Descriptives (Note: some of the respondents failed to report their age. Some of the respondents never reported discomfort or pain levels of 8/10).

	N	Mean	SD	Min	Max
Age (years)	51	26.0	4.7	21	44
Water temperature before task (°C)	54	0.8	0.6	0	2.5
Water temperature after task (°C)	54	0.3	0.5	0	2.5
Cold pressor task pain threshold (s)	54	18.5	15.1	0	76.4
Cold pressor task total time (s)	54	65.7	58.3	12.6	180+
Cold pressor task pain 8/10 (s)	51	29.9	30.1	5.30	171.6
Maximum grip strength 1 (kg)	54	27.6	7.56	13	48
Maximum grip strength 2 (kg)	54	27.9	6.85	12	43
Hand grip endurance task 1 (s)	54	96.0	47.5	17.2	180+
Hand grip endurance task 2 (s)	54	76.6	38.4	18.9	180+
Hand grip endurance task 1 discomfort 8/10 (s)	45	49.1	37.1	11.3	160.5
Hand grip endurance task 2 discomfort 8/10 (s)	46	48.1	31.2	7	157.5
Verbal reasoning tasks correct (amount)	54	6.80	2.73	1	11
Verbal reasoning tasks attempted (amount)	54	10.6	3.26	3	14
Verbal reasoning task (points)	54	10.5	4.51	1	17
Impossible verbal reasoning task time (s)	54	98.9	65.0	10.4	299.1
Impossible anagram task time (s)	54	54.1	39.8	6.89	196.8
Anagram tasks correct (amount)	54	4.80	2.76	1	13
Anagram tasks attempted (amount)	54	10.6	4.31	4	18
Thread and needle task (s)	54	75.9	76.2	6.8	300+
Boring video task (s)	54	99.4	67.0	9.2	300+

**Table 2:** Loadings for rotated factor matrix: extraction by Maximum Likelihood method. Factor 1 could be described as “physical BP” and factor 2 could be described as “mental BP”.

	Factor	
	1	2
Hand grip endurance task time	0.778	
Cold pressor task time	0.746	
Impossible verbal reasoning task time	0.143	0.604
Thread and needle task time	0.428	0.466
Boring video task time	0.262	0.349
Impossible anagram task time		0.295

**Table 3:** Eigenvalues and the cumulative percentage explained by the factors.

Factor	Initial Eigenvalues			Extraction Sums of Squared Loadings			Rotation Sums of Squared Loadings		
	Total	% of Variance	Cumulative %	Total	% of Variance	Cumulative %	Total	% of Variance	Cumulative %
1	2.19	36.5	36.5	1.6	27.4	27.4	1.44	24.0	24.0
2	1.23	20.5	57.0	0.60	9.9	37.3	0.80	13.3	37.3

**Table 4:** Correlations between factor variables and tasks performances.

	1.	2.	3.	4.	5.	6.	7.	8.	9.	10.
1. Factor 1 “physical” BP ^1^	1									
2. Factor 2 “mental” BP ^2^	0.142	1								
3. Cold pressor task time	0.862**	0.035	1							
4. Hand grip endurance task time 1	0.900 **	0.122	0.584 **	1						
5. Hand grip endurance task time 2	0.697 **	0.012	0.424 **	0.813 **	1					
6. Impossible verbal reasoning task time	0.165	0.839 **	0.12	0.166	0.039	1				
7. Impossible anagram task time	−0.081	0.409 **	−0.035	−0.03	−0.011	0.18	1			
8. Thread and needle task time	0.495 **	0.647 **	0.336 *	0.364 **	0.195	0.358 **	0.051	1		
9. Boring video task time	0.303 *	0.484 **	0.191	0.248	0.22	0.211	0.159	0.287 *	1	
10. “Physical” BP sum ^3^	0.956 **	0.341 *	0.813 **	0.826 **	0.607 **	0.273 *	−0.006	0.721 **	0.308 *	1
11. “Mental” BP sum ^4^	0.400 **	0.902 **	0.277 *	0.322 *	0.142	0.824 **	0.14	0.824 **	0.302 *	0.603 **

* *p* < 0.05; ** *p* < 0.01. ^1.^ Factor 1 “physical” BP and ^2.^ Factor 2 “mental” BP are BP-related variables that have been formed by creating a combined factor variable based on the actual factor loadings. ^3^ The “Physical” BP sum has been formed by adding together the cold pressor task time and hand grip endurance task time. ^4^ The “Mental” BP sum has been formed by adding together the impossible verbal reasoning task time and thread and needle task time.

**Table 5:** Relative effect sizes for separate variables. One analysis is presented per row, “BP trait” and “depletion” variables were used to explain the performances in the tasks in the left column.

	Performance in Other Tasks, “BP Trait”		Rank Order Variable, “Depletion”	
	*η* ^2^	p	*η* ^2^	p
Cold pressor task	0.188	<0.01	0.089	0.037
Hand grip endurance task	0.253	<0.001	0.001	0.83
Impossible verbal reasoning task	0.221	<0.001	0.096	0.030
Impossible anagram task	0.011	0.47	0.091	0.036
Thread and needle task	0.170	<0.01	0.019	0.35
Boring video task	0.131	<0.01	-	-

## Supplementary Materials

The following are available online at www.mdpi.com/article/10.3390/bs11090123/s1, Table S1: Loadings for unrotated Factor Matrix: Extraction by Maximum Likelihood-method, Table S2: Partial correlation between sum-variables based on factor analysis and physical attributes; all analysis controlled for sex, Table S3: Descriptives of interbeat intervals (IBI) in different tasks in milliseconds (rounded to the nearest millisecond), Table S4: Correlations between interbeat intervals (IBI) in different tasks, Table S5: Correlations between traits (i.e. factor variables and corresponding sum variables) and interbeat intervals (IBI) during different tasks.

## Abbreviations

BP	Behavioural perseverance or persistence
IBI	Interbeat interval
HRV	Heart rate variability
