# Supplementary material for: Evidence for a Behaviourally Measurable Perseverance Trait in Humans
Source: Behav Sci (Basel). 2021 Sep 9;11(9):123. doi: 10.3390/bs11090123 (PMC8468401; doi:10.3390/bs11090123)
Supplement: Supplementary file 1 [file behavsci-11-00123-s001.zip › behavsci-1318786-supplementary.pdf]

Supplementary Table S1. Loadings for unrotated Factor Matrix: Extraction by Maximum Likelihood-method

	Factor	
	1	2
Hand grip endurance task time	0.765	-0.168
Cold pressor task time	0.714	-0.217
Impossible verbal reasoning task time	0.330	0.526
Thread and needle task time	0.556	0.303
Boring video task time	0.361	0.245
Impossible anagram task time		0.302

Supplementary Table S2. Partial correlation between sum-variables based on factor analysis and physical attributes; all analysis controlled for sex

	1	2	3	4	5
1. "Physical" BP sum[1]	1				
2. "Mental" BP sum[2]	0.48**	1			
3. Hand grip strength	0.31*	0.17	1		
4. Age	0.12	0.08	0.35*	1	
5. Height	-0.04	0.04	0.34*	-0.28	1
6. Weight	0.26	0.21	0.44**	0.04	0.55***

*p<0.05; **p<0.01; ***p<0.001

[1] "Physical" sum has been formed by adding together cold pressor task time and hand grip endurance task time.

[2] "Mental" sum has been formed by adding together impossible verbal reasoning task time and thread and needle task time.

Supplementary Table S3. Descriptives of interbeat intervals (IBI) in different tasks in milliseconds (rounded to the nearest millisecond)

	N	Mean	SD	Min	Max
Cold pressor task IBI	53	727	130	519	1194
Hand grip endurance task IBI	54	682	139	480	1106
Thread and needle task IBI	54	837	145	564	1186
Verbal reasoning task IBI	53	851	152	591	1269
Anagram task IBI	54	852	158	618	1285
Boring video task IBI	54	931	171	634	1385

Supplementary Table S4. Correlations between interbeat intervals (IBI) in different tasks

	1.	2.	3.	4.	5.
1. Boring video task IBI	1				
2. Cold pressor task IBI	0.82**	1			
3. Hand grip endurance task IBI	0.78**	0.67**	1		
4. Thread and needle task IBI	0.92**	0.79**	0.80**	1	
5. Impossible verbal reasoning task time IBI	0.96**	0.81**	0.77**	0.91**	1
6. Impossible anagram task IBI	0.96**	0.78**	0.76**	0.91**	0.91**

**p<0.01

Supplementary Table S5. Correlations between traits (i.e. factor variables and corresponding sum variables) and interbeat intervals (IBI) during different tasks.

	Factor 1 "physical" BP	Factor 2 "mental" BP	"Physical" BP sum[1]	"Mental" BP s[illegible]
Cold pressor task IBI	0.49***	-0.15	0.44**	0.04
Hand grip endurance task IBI	0.38**	-0.05	0.33*	0.05
Verbal reasoning task IBI	0.35*	-0.08	0.31*	0.02
Anagram task IBI	0.21	-0.02	0.19	0.04
Thread and needle task IBI	0.31*	-0.07	0.30*	0.05
Boring video task IBI	0.34*	-0.04	0.31*	0.04

*p<0.05; **p<0.01; ***p<0.001

[1] “Physical” BP sum has been formed by adding together cold pressor task time and hand grip endurance task time.

[2] “Mental” BP sum has been formed by adding together impossible verbal reasoning task time and thread and needle task time.
